# Patient Perspectives After Trapeziectomy Versus Carpometacarpal Prosthesis: A Qualitative Thematic Analysis of Ten Bilateral Cases

**DOI:** 10.3390/jcm14238375

**Published:** 2025-11-26

**Authors:** Léna G. Dietrich, Valeria Rinaldi, Esther Vögelin

**Affiliations:** Department of Plastic and Hand Surgery, Inselspital University Hospital Bern, University of Bern, 3010 Bern, Switzerland; valeria.rinaldi@students.unibe.ch (V.R.); esther.voegelin@insel.ch (E.V.)

**Keywords:** carpometacarpal arthritis, trapeziectomy, thumb prosthesis, qualitative research, patient perspectives, functionality, aesthetics, rehabilitation, psychosocial impact, shared decision-making

## Abstract

**Background**: Carpometacarpal (CMC-I) arthritis is a frequent and disabling condition. Standard surgical options include trapeziectomy and prosthetic arthroplasty. While quantitative outcomes have been widely studied, little is known about patient perspectives regarding function, aesthetics, and rehabilitation. **Methods**: We conducted semi-structured interviews with ten patients who had undergone trapeziectomy on one side and prosthesis implantation on the contralateral side. Interviews were performed ≥6 months postoperatively, audio-recorded, transcribed verbatim, and analyzed thematically following Braun and Clarke’s framework. Researcher triangulation and member checking were applied to enhance trustworthiness. **Results**: Four overarching themes were identified. (1) Strength: Most patients reported greater strength and endurance on the prosthetic side, though both hands were generally adequate for daily activities. (2) Rehabilitation: Recovery after prosthesis implantation was described as markedly faster and less burdensome, with reduced need for therapy compared to trapeziectomy. (3) Aesthetics: Trapeziectomy was often associated with dissatisfaction due to thumb shortening and collapse, while prostheses were perceived as restoring a more natural appearance. (4) Surgical preference: When asked which procedure they would hypothetically choose again, all participants favored prosthesis implantation, citing superior function, faster recovery, and more favorable aesthetics. **Conclusions**: Patients who experienced both procedures consistently preferred prosthesis implantation. Their narratives highlight dimensions beyond standard clinical scores, including rehabilitation burden, appearance, and psychosocial impact. Incorporating patient-reported outcomes into surgical counseling is essential to align treatment of CMC-I arthritis with patient priorities and to support shared decision-making.

## 1. Introduction

Carpometacarpal (CMC-I) arthritis of the thumb is one of the most common causes of pain and disability in the hand [1]. Recent population-based studies report a prevalence of approximately 7–15% among middle-aged and elderly individuals, with a marked predominance in postmenopausal women [2,3]. Surgical options have evolved over decades, beginning with trapeziectomy as first described by Gervis in 1947 [4] and later the development of total joint replacements, pioneered by de la Caffinière in 1974 [5]. Contemporary meta-analyses demonstrate transient short-term benefits of total joint arthroplasty in pain and function up to 3 months, but no sustained advantage at 1 year compared with trapeziectomy [2]. A recent review of reviews further emphasized persistent methodological limitations in existing meta-analyses and the absence of definitive evidence favoring any single surgical technique [6]. This finding highlights the ongoing debate between early functional recovery and long-term implant durability.

Trapeziectomy, with or without ligament reconstruction and tendon interposition, has long been considered the gold standard [7]. Nevertheless, multiple comparative studies have suggested that prosthetic arthroplasty may provide advantages in terms of grip strength, faster rehabilitation, and patient satisfaction [8,9,10,11,12]. Recent prospective comparative research confirmed these findings, showing significantly faster return to work and earlier functional recovery after prosthesis implantation without increased short-term complication rates [13]. Most of these investigations, however, rely primarily on quantitative outcomes and complication rates [14,15,16].

Beyond these quantitative measures, variability in postoperative rehabilitation and therapy regimens substantially shapes patient experience [17]. Reported immobilization duration and use of hand therapy vary widely, reflecting the absence of standardized protocols.

We recently reported a same-patient side-by-side comparison of trapeziectomy and contralateral CMC-I prosthesis [18]. That study demonstrated clear advantages of prosthesis implantation with respect to strength, therapy duration, pain relief, and aesthetic outcomes, and all patients indicated a preference for prosthesis when asked about a hypothetical third hand. In parallel, online patient education on thumb CMC arthroplasty remains limited and difficult to comprehend for laypersons [19]. Given that health literacy strongly influences satisfaction, decision confidence, and adherence to postoperative recommendations, understanding patient perspectives beyond clinical metrics becomes even more important. While these findings confirmed alignment between objective improvements (grip strength, recovery time) and subjective preferences, the analysis was limited by its concise format and relied on descriptive quantitative results complemented by only selected quotations.

In contrast, the present work expands on these findings through a systematic qualitative approach. By conducting and thematically analyzing in-depth semi-structured interviews, we aimed to capture a richer spectrum of patient experiences, including functional recovery, resilience, aesthetic perceptions, and the decision-making process. By complementing quantitative evidence and capturing domains often underrepresented in traditional outcome studies, this approach fosters a more nuanced understanding of patient perspectives and emphasizes the importance of integrating subjective experiences into a multidimensional framework for outcome assessment in hand surgery and rehabilitation.

Qualitative research has proven particularly useful to capture lived experiences of recovery, coping, and adaptation in hand surgery [20,21]. Such approaches elucidate how psychosocial and contextual factors influence rehabilitation and perceived surgical success. Moreover, patient-derived frameworks for quality emphasize that communication, expectation management, and trust are central to patients’ perceived treatment value [22,23]. These insights collectively support the need for patient-centered evaluation and justify our qualitative exploration of patient perspectives after CMC arthroplasty and trapeziectomy.

## 2. Methods

### 2.1. Study Design

We conducted a qualitative, exploratory interview study to gain an in-depth understanding of patient perspectives after trapeziectomy and contralateral carpometacarpal (CMC-I) prosthesis implantation. This study represents a qualitative analysis of the same cohort of ten bilateral patients previously reported [18]. The present work, however, applies an independent qualitative design and thematic analysis to explore patient perspectives in greater depth. Qualitative methodology was chosen to capture subjective experiences that are not easily assessed by standardized questionnaires or quantitative outcomes. Following methodological approaches in recent qualitative hand-surgery studies [20,21], semi-structured interviews explored patients’ lived experiences, recovery challenges, and perceived function in everyday activities. This qualitative study is reported in accordance with the Consolidated Criteria for Reporting Qualitative Research (COREQ) 32-item checklist (Tong et al., 2007 [24], Supplementary File S1). Particular attention was given to transparency in sampling and recruitment, the interviewer’s role and training, the transcription and coding process, and procedures to enhance trustworthiness, including triangulation and member checking.

### 2.2. Participants and Recruitment

**Figure 1:**
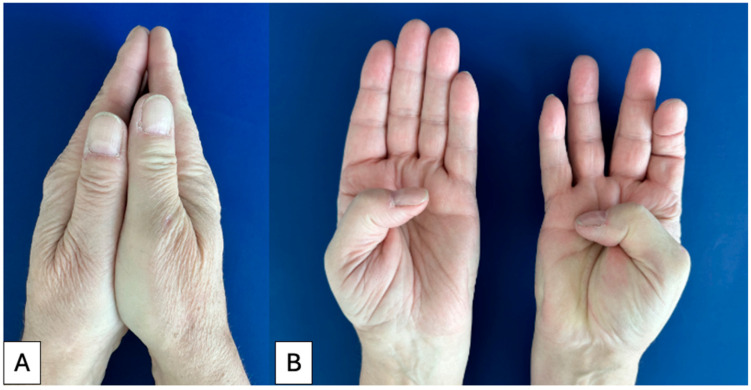
Clinical photographs of a male patient with left trapeziectomy and right carpometacarpal prosthesis. Male patient after left trapeziectomy and right prosthesis implantation, showing visible thumb length difference (**A**) and almost symmetrical opposition (**B**).

Ten patients who had undergone both procedures between 1999 and 2023 were invited to participate (Table 1, Figure 1). Eligible patients were identified through institutional surgical records and contacted by mail or telephone. Inclusion criteria were (i) history of trapeziectomy on one side and prosthesis implantation on the contralateral side, (ii) minimum of six months elapsed since the second operation to allow functional recovery, and (iii) willingness and ability to participate in an interview in German. Follow-up durations differed substantially between sides: trapeziectomy cases ranged from 6.6 to 25 years (mean ≈ 11 years), while prosthesis cases ranged from 0.7 to 6.9 years (mean ≈ 4 years). All prostheses were ball-and-socket, dual-mobility-type implants (Maïa^®^ or Touch^®^), implanted according to the manufacturer’s standard technique. Trapeziectomy was performed using the standard technique with tendon suspension and interposition (LRTI) with a flexor carpi radialis tendon slip, applied consistently across all cases. No explicit exclusion criteria were applied beyond inability to provide informed consent. Demographic information such as age, sex, and hand dominance was collected and summarized (Table 1). Quantitative outcomes from this cohort have previously been reported [18]. The present study provides a distinct, qualitative analysis of patient interviews, focusing on thematic evaluation of patient perspectives, resilience, aesthetics, and shared decision-making. Follow-up duration differed markedly between procedures (trapeziectomy 6.6–25 years, mean ≈ 11 years; prosthesis 0.7–6.9 years, mean ≈ 4 years). Because longer follow-up can influence perceived endurance and late satisfaction, this asymmetry was considered during coding and interpretation.

### 2.3. Data Collection

Semi-structured interviews were conducted in person by the first author (L.G.D.), a medical doctor trained in qualitative research methods. She was not the operating surgeon, thereby reducing potential dependency bias. To accommodate patient preferences, interviews took place either at the hospital or at the participants’ homes. Each interview lasted between 25 and 60 min and was conducted in German. Quotations presented in this article were translated into English for publication. Translations were cross-checked by bilingual members of the research team to ensure conceptual accuracy and fidelity.

An interview guide was used to ensure consistency across interviews while allowing participants to elaborate freely (Supplementary File S2). Topics included postoperative pain, duration and burden of rehabilitation, strength and endurance, functionality in daily life and work, aesthetics, and surgical preferences. Patients were explicitly asked to compare their experiences with both surgical techniques and to indicate which procedure they would hypothetically prefer for a third hand. Interview style was conversational, and the interviewer encouraged participants to provide detailed narratives and concrete examples.

All interviews were audio-recorded with participant consent, transcribed verbatim by the interviewer, and anonymized. Notes on contextual factors (e.g., setting, non-verbal cues) were added to enrich interpretation.

### 2.4. Data Analysis

We applied thematic analysis as described by Braun and Clarke [25]. Coding and theme development were performed iteratively and reflexively, as recommended for patient-centered qualitative studies [21]. The analysis also considered potential researcher bias through reflexive journaling and team triangulation. The analysis followed six iterative steps: (1) familiarization with the data through repeated reading of transcripts, (2) systematic generation of initial codes, (3) collation of codes into preliminary themes, (4) review of themes against the data set, (5) definition and naming of themes, and (6) production of the report with illustrative quotations. The coding process followed an iterative and reflexive approach. Because the sample was small and heterogeneous, we applied the concept of thematic sufficiency rather than claiming full saturation, focusing on identifying recurring patterns across interviews. Themes and subthemes identified from patient interviews are shown in Table 2.

Two authors independently coded the material by hand (Supplementary File S3). Coding differences were discussed until consensus was reached. To enhance transparency, we applied elements of the Framework Method [26], organizing data in comparative matrices that allowed systematic cross-case analysis. Trustworthiness of the analysis was enhanced by several strategies: investigator triangulation through peer debriefing within the research team, member checking in which patients reviewed summaries of their transcripts, and documentation of analytic decisions to ensure confirmability.

### 2.5. Researcher Reflexivity

The interviewer (L.G.D.) had prior experience in qualitative interviewing and maintained a neutral stance toward both surgical techniques. As she was not the operating surgeon for any participant, potential influence through treatment dependency was minimized. Reflexive notes were taken during and after each interview to account for the researcher’s perspective in the analytic process.

### 2.6. Ethics

The study was conducted in accordance with the principles of the Declaration of Helsinki and institutional policies. All participants provided written informed consent for participation and publication of anonymized quotations using the institutional general consent form.

## 3. Results

We identified four overarching themes emerging from the interviews: strength, resilience and rehabilitation, aesthetics, and surgical preferences. Each of the four themes was mentioned by at least eight of the ten participants, indicating strong thematic consistency across interviews. Despite individual variations, thematic overlap was high across participants. Women tended to elaborate more on aesthetic appearance and rehabilitation experiences, while men more often emphasized strength and everyday functionality. Dominance side did not appear to decisively influence satisfaction, though patients often mentioned increased confidence when the prosthetic side was dominant. These themes mirror domains described in prior qualitative work emphasizing recovery trajectory, psychosocial adjustment, and the influence of information on daily life [21,22]. Follow-up durations are summarized in Table 1 and differed markedly between sides (trapeziectomy 6.6–25 years, mean ≈ 11 years; prosthesis 0.7–6.9 years, mean ≈ 4 years). When interpreting patient statements related to endurance and long-term satisfaction, this difference in follow-up duration was explicitly considered to avoid over-attributing perceived endurance advantages to the type of procedure alone.

### 3.1. Strength

Patients expressed heterogeneous perceptions of strength after surgery. While both techniques led to improvement compared to the preoperative state, many participants perceived the prosthesis side as stronger and more enduring. One male participant reflected: “I am aware that I have two operated thumbs. It is unrealistic to speak of a healthy hand here; there is strength loss on both sides. But I would describe it as minor on the prosthetic side and major on the trapeziectomy side.” A female patient emphasized the gain in strength through pain relief: “Before surgery, I had hardly any strength because of arthritis. After both operations, I improved, but my prosthetic thumb is noticeably stronger—and it also happens to be my dominant side.” Others stressed functional adequacy rather than differences: “I’m already old—both sides are perfectly sufficient for my daily activities,” reported a female participant.

“In terms of everyday function, I don’t notice a relevant difference; I cannot say one side is better than the other,” noted another female participant. Nevertheless, fatigue differences were consistently reported. A male participant stated: “Both operations gave me more strength, but the trapeziectomy side tires faster. Sometimes I even have to switch to my weaker left hand.” These statements highlight that while both operations restore strength, prosthesis implantation was more often associated with endurance and confidence in daily tasks. Several participants described a renewed sense of trust in the prosthetic thumb during physically demanding activities such as gardening, opening jars, or carrying bags. Conversely, the trapeziectomy side was perceived as ‘trustworthy but slower’, often requiring deliberate caution during heavy tasks. This perceived reliability was described as equally physical and psychological, reflecting regained security in daily actions.

### 3.2. Resilience and Rehabilitation

Resilience and rehabilitation requirements were described as markedly different. Prosthesis implantation was consistently associated with a shorter, less burdensome rehabilitation period. One male participant explained: “After the prosthesis, I needed far less occupational therapy, which had much less impact on my daily life. After the trapeziectomy, I attended therapy for almost half a year.” Female patients confirmed this contrast: “I was surprised how quickly I recovered after the prosthesis. Only three therapy sessions, and I was back to normal.”

“After the trapeziectomy, I had to relearn almost everything in therapy—my coordination was really poor.” Long-term differences were also noted. A female participant noted: “Even years later, I can use both hands well, but the trapeziectomy hand clearly sets the limits for carrying weight over time.” The reduced therapy demand after prosthesis was often linked to improved quality of life. Participants also mentioned the social impact of the rehabilitation phase, longer therapy after trapeziectomy restricted participation in hobbies or community activities, while shorter rehabilitation after prosthesis allowed earlier reintegration into social life: “With the prosthesis, I was back to cooking, shopping, and looking after my grandchildren much sooner. The trapeziectomy slowed me down for months,” reported a female participant.

Participants consistently associated rehabilitation with patience, discipline, and the support of occupational therapy. Some described the process as a test of endurance, while others emphasized emotional adaptation as equally important as physical recovery. Those who recovered more rapidly reported a sense of independence and pride, frequently linking this to their ability to resume household or caregiving roles.

### 3.3. Aesthetics

Aesthetic outcomes emerged as a dominant theme, particularly among female participants. Trapeziectomy was frequently associated with dissatisfaction due to thumb shortening, collapse, or swelling. One female participant noted: “For me, the sunken thumb is aesthetically unappealing. It’s complaining at a high level, but before surgery I didn’t realize how much it would bother me.” Another female participant added: “The difference in length is not the main problem, it is the doughy swelling on the trapeziectomy side. I prefer the prosthetic thumb and tend to hide the other one.” In contrast, prosthesis results were often described as natural: “The thumb with the prosthesis feels much more like a normal thumb. I completely forget that surgery even took place,” reported a male participant. Male participants noticed differences but were less concerned: “The trapeziectomy side looks different, yes, but it doesn’t disturb me. The prosthesis thumb just seems more like before.” No participant expressed negative aesthetic comments regarding the prosthesis. Scars were not seen as problematic. However, some participants described subtle sensory differences, such as reduced soft-tissue pliability or stiffness at the trapeziectomy scar, which occasionally reminded them of surgery. For some, aesthetic perception was strongly tied to overall satisfaction: “I simply like my thumb with the prosthesis better, I would rather hide the other one,” reported a female participant.

Aesthetic concerns extended beyond appearance to body image and identity. For several participants, the trapeziectomy thumb symbolized “aging hands”, while the prosthetic thumb was associated with normality and restored confidence. Younger female participants were particularly sensitive to visible asymmetry, whereas older participants described a pragmatic acceptance.

### 3.4. Surgical Preferences

When asked which technique they would prefer for a hypothetical “third hand”, all patients favored the prosthesis, citing superior function, faster recovery, and better appearance. One female patient remarked: “I would choose the prosthesis again without hesitation. It looks more aesthetically pleasing, whereas the side with the tendon sling collapses and sometimes even goes numb.” Another male patient highlighted recovery: “I think both techniques are good, and I don’t notice a huge difference. Still, I would choose the prosthesis, because the recovery period was significantly shorter.” A third (male) emphasized reversibility: “Why should I choose a solution with no point of return, when there is a reliable alternative?” Together, these unanimous responses underscore that, although trapeziectomy is effective, patients consistently associated prosthesis implantation with outcomes that mattered most to them in daily life. Even when acknowledging the prosthesis as an artificial device, participants expressed strong trust in its performance and reliability. Decisions were portrayed as pragmatic rather than emotional: the majority highlighted time efficiency, independence, and confidence as decisive factors, while only a few mentioned concerns about implant wear or revision.

In summary, participants described both surgical options as effective in alleviating pain and restoring daily function, yet differentiated them according to dimensions that mattered most in daily life. Prosthesis implantation was consistently associated with strength, confidence, and aesthetics, while trapeziectomy was perceived as reliable but limited in endurance and appearance. Rehabilitation time, aesthetic satisfaction, and sense of normality emerged as decisive factors shaping perceived success.

## 4. Discussion

This qualitative study provides novel insights into patient perspectives after trapeziectomy and contralateral prosthesis implantation and is methodologically and conceptually distinct from our earlier quantitative report. Whereas most previous research has focused on objective outcomes such as grip strength, complication rates, and implant survival [8,10,15], our findings highlight subjective dimensions—rehabilitation burden, aesthetic perception, and surgical preferences, that strongly influenced satisfaction. These results complement our earlier quantitative analysis of the same cohort [18] and expand the evidence base by systematically capturing lived experiences through thematic analysis.

In addition to clinical findings, this study illustrates the value of qualitative methodology in surgical outcome research. Standardized scores capture only part of the patient experience, while interviews reveal perspectives on endurance, aesthetics, and daily life that are rarely visible in quantitative frameworks. This approach extends our earlier report [18], which documented functional differences but provided limited contextualization of patient narratives. By embedding numerical results within a patient-centered framework, we offer a deeper understanding of why prostheses were unanimously preferred. Our findings align with Liukkonen et al. (2024) [2], who noted that early functional benefits after arthroplasty may explain patients’ subjective preference for prosthetic reconstruction despite uncertain long-term outcomes.

The paired design represents a particular strength, allowing within-patient comparisons and reducing inter-individual variability. Such designs are rare in hand surgery and provide robust insights into patient preferences. Methodological rigor was further supported by triangulation, member checking, and systematic coding.

### 4.1. Strength and Function

Patients generally reported superior strength and endurance on the prosthetic side, while trapeziectomy outcomes were perceived as functional but less robust and prone to quicker fatigue. These perceptions are consistent with quantitative reports of improved pinch and grip strength after prosthetic arthroplasty [9,11], and echo our prior finding of higher measured grip strength with prostheses [18]. Importantly, patients emphasized the everyday significance of endurance, such as the ability to carry groceries or perform repetitive tasks, underscoring that functional adequacy is not only about peak force but also about sustained performance.

### 4.2. Rehabilitation and Resilience

The contrast in rehabilitation burden was among the most striking findings. Patients described recovery after trapeziectomy as long and demanding, often requiring months of therapy and relearning of coordination, whereas rehabilitation after prosthesis was typically short and minimally disruptive. These accounts corroborate prospective studies showing faster recovery with prostheses [27]. Importantly, patients linked shorter therapy to broader life impact, noting faster return to work, family responsibilities, and leisure activities. Even at long-term follow-up for trapeziectomy and mid-term follow-up for prosthesis cases, patients still perceived the trapeziectomy side as less resilient with limitations in weight-bearing capacity. Comparable trends of faster functional recovery and higher patient satisfaction after CMC prosthesis implantation were also reported by Smeraglia et al. [28].

### 4.3. Aesthetic Perceptions

Aesthetic outcomes emerged as a central and sometimes unexpected determinant of satisfaction, particularly for female participants. Trapeziectomy was frequently described as leading to thumb shortening, collapse, or swelling, which patients found disturbing. In contrast, prostheses were perceived as restoring a natural appearance, with several patients stating that they “forgot surgery had taken place.” This dimension is rarely reported in outcome studies but resonates with isolated observations of cosmetic dissatisfaction after trapeziectomy [15]. Our findings suggest that appearance is not merely a secondary consideration but a core component of patient well-being and satisfaction, with potential psychosocial implications.

### 4.4. Patient Preferences and Shared Decision-Making

All participants favored prosthesis implantation for a hypothetical ‘third hand’, primarily due to faster recovery, greater perceived strength, and more favorable aesthetics. Although many patients viewed both techniques as effective, prostheses were consistently aligned with what they considered most relevant in daily life—independence, endurance, and natural appearance. These findings echo the same-patient study by Nietlispach et al. [29], and highlight the importance of integrating patient-reported priorities into preoperative counseling. Beyond clinical scores, shared decision-making should therefore address rehabilitation burden and aesthetic expectations, as these dimensions strongly shaped patient satisfaction in our cohort.

### 4.5. Long-Term Experiences

Several participants perceived the prosthetic side as stronger and more reliable, despite shorter follow-up compared with the trapeziectomy side. This impression is consistent with reports of stable mid-term outcomes and good implant survival of modern dual-mobility designs [9,15,30,31]. In contrast, trapeziectomy was described as durable but limited in endurance and appearance—an observation congruent with long-term data showing reliable pain relief but reduced strength after resection arthroplasty [7]. Recent biomaterials research has suggested that advanced surface coatings and improved biocompatibility may enhance implant longevity [32]. Hydroxyapatite- and titanium-coated surfaces, for example, have been shown to promote osteointegration and mechanical stability of thumb implants [33]. In parallel, emerging regenerative strategies such as mesenchymal stem cell–derived extracellular vesicles offer potential adjuncts for joint repair [32], while single-cell transcriptomic analyses continue to refine the molecular understanding of osteoarthritis and may inform future implant and tissue-engineering developments [34].

### 4.6. Complications and Revision Concerns

Patients expressed awareness of potential implant-related risks, but no major complications were reported in our cohort. Their risk perception appears consistent with multicenter data showing low early failure and complication rates for modern implants [35]. Earlier prosthesis generations, such as the Elektra [9,31] or Roseland [36] designs, were associated with higher failure rates [37,38], whereas modern dual-mobility implants show improved outcomes and lower revision rates [16,39]. Some patients perceived the possibility of secondary trapeziectomy as a safety net, an impression supported by studies demonstrating that outcomes after secondary trapeziectomy following implant revision are comparable to those of primary trapeziectomy [40].

### 4.7. Doctor–Patient Communication and Expectations

Several patients reflected critically on preoperative counseling. Some felt insufficiently informed about possible aesthetic changes after trapeziectomy, which later became a source of dissatisfaction. This underscores the importance of comprehensive shared decision-making that includes discussion of not only pain relief and functional expectations but also cosmetic and psychosocial outcomes. Transparent communication is essential for aligning surgical recommendations with patient values, a principle emphasized in recent qualitative work on decision-making in hand surgery [29].

### 4.8. Quality of Life and Psychosocial Impact

Patient narratives frequently linked surgical outcomes to broader aspects of daily life, such as independence, ability to maintain hobbies, and social confidence. Prosthesis implantation was often associated with “feeling normal again” or “forgetting the disease”, while trapeziectomy occasionally left visible or functional reminders. These findings highlight the psychosocial weight of outcomes, which is rarely captured by standard scores but is critical for patient-centered evaluation.

### 4.9. Strengths and Limitations

The main strength of this study lies in its unique paired design, which enabled within-patient comparisons and minimized inter-individual variability. Additional strengths include systematic thematic analysis, investigator triangulation, and member checking, all of which enhanced rigor and credibility. Limitations include the small sample size, retrospective design, and single-center setting, which may restrict generalizability. Although recurring patterns were identified, the small sample size, particularly in the male subgroup, does not allow us to claim full thematic saturation. Instead, our findings reflect thematic sufficiency within this specific cohort. A further limitation concerns the asymmetry of follow-up durations: trapeziectomy sides included cases with up to 25 years of follow-up, whereas prosthesis sides had shorter, mid-term observation periods (mean ≈ 4 years, range 0.7–6.9 years). This temporal imbalance may have influenced perceptions of endurance, pain, or aesthetics. Given the small sample size and heterogeneity in follow-up duration, these findings should be interpreted with caution. Because included cases span more than two decades (1999–2023), recall bias and evolution of surgical techniques may have affected patient recollection and perceived outcomes.

### 4.10. Clinical Implications

Our findings emphasize that surgical decision-making should not be guided solely by complication rates or implant survival. Rehabilitation burden, aesthetic outcomes, and psychosocial dimensions strongly shaped patient satisfaction in our cohort. Prosthetic arthroplasty may represent an attractive option for carefully selected patients, particularly for those who prioritize rapid recovery and appearance, while decisions should remain individualized and evidence-informed. Future research should integrate mixed-methods approaches to combine quantitative and qualitative insights, ensuring that patient-centered outcomes inform both clinical guidelines and individual decision-making.

### 4.11. Limitations and Future Directions

This qualitative study was limited by its small, single-center sample, yet offers rare same-patient comparisons. Future research should integrate patient-reported outcome measures and qualitative feedback in mixed-methods designs to advance value-based hand care [22,23]. Expanding this approach could support development of decision-aids and individualized rehabilitation programs.

## 5. Conclusions

This qualitative study provides novel, in-depth insights into patient experiences after trapeziectomy and contralateral carpometacarpal prosthesis implantation. Patients who had undergone both procedures consistently described faster recovery, greater perceived strength, and more favorable aesthetics following prosthesis implantation, while trapeziectomy was often associated with longer rehabilitation and aesthetic dissatisfaction. These findings highlight that perceived success extends beyond clinical scores and depends strongly on daily functionality, rehabilitation burden, and self-image.

While all participants favored the prosthetic side, these observations should be interpreted as exploratory and hypothesis-generating rather than prescriptive. The qualitative nature and small, single-center sample preclude generalization, and time-related factors, such as markedly longer follow-up for trapeziectomy (up to 25 years) versus mid-term follow-up for prostheses (mean ≈ 4 years, 0.7–6.9 years), likely influenced perceptions of endurance and appearance. Likewise, recall bias and surgical evolution over two decades must be acknowledged.

Nevertheless, by directly comparing both techniques within the same individuals, this study uniquely contextualizes quantitative outcomes with personal narratives. The results emphasize the importance of transparent preoperative counseling, expectation management, and integration of patient-reported experiences into shared decision-making. Future research should adopt mixed-methods approaches combining qualitative insights with standardized outcomes and long-term implant data to inform evidence-based and patient-centered management of CMC-I arthritis.

## Figures and Tables

**Table 1:** Demographic and surgical characteristics of the study cohort (*n* = 10).

	Gender	Age	Time Since Surgery (Years) Trapeziectomy	Time Since Surgery (Years) Prosthesis	Dominant Hand
Patient 1	female	75	25 (right)	3.8 (left)	right
Patient 2	female	68	7.8 (left)	6.9 (right)	right
Patient 3	male	64	7.6 (left)	4.9 (right)	left
Patient 4	female	76	6.6 (left)	6.5 (right)	right
Patient 5	male	70	14.7 (left)	6.8 (right)	right
Patient 6	female	68	9.4 (left)	5.8 (right)	right
Patient 7	female	63	11.6 (right)	0.8 (left)	right
Patient 8	male	73	14 (left)	2.9 (right)	left
Patient 9	female	77	8.8 (right)	1.1 (left)	right
Patient 10	female	74	7.6 (right)	0.7 (left)	right

**Table 2:** Themes and subthemes identified from patient interviews.

Theme	Subthemes	Illustrative Quotations
Strength	Perceived stronger prosthesis side; Adequacy for daily tasks; Fatigue differences	“My prosthetic thumb is noticeably stronger.” (female) “Both sides are sufficient for daily life.” (female) “The trapeziectomy side tires faster.” (male)
Rehabilitation and resilience	Faster recovery with prosthesis; Reduced therapy burden; Long-term endurance differences	“After the prosthesis, I needed far less therapy.” (male) “Only three therapy sessions, and I was back to normal.” (female) “The trapeziectomy hand sets the limits for carrying weight.” (female)
Aesthetics	Dissatisfaction after trapeziectomy (shortening, collapse, swelling); Natural appearance with prosthesis; Psychosocial impact	“The sunken thumb is aesthetically unappealing.” (female) “The prosthesis feels like a normal thumb.” (male) “I prefer the prosthetic thumb and hide the other one.” (female)
Surgical preference	Hypothetical choice for prosthesis; Faster recovery; Reversibility and reliability	“I would choose the prosthesis again without hesitation.” (female) “Recovery was significantly shorter with prosthesis.” (male) “Why choose a solution with no point of return?” (male)

## Data Availability

Dataset is available on request from the authors.

## Supplementary Materials

The following supporting information can be downloaded at: https://www.mdpi.com/article/10.3390/jcm14238375/s1, File S1: COREQ 32-item checklist; File S2: Interview guide (English translation); File S3: Coding matrix/theme overview.
