# Supplementary material for: Patient Perspectives After Trapeziectomy Versus Carpometacarpal Prosthesis: A Qualitative Thematic Analysis of Ten Bilateral Cases
Source: J Clin Med. 2025 Nov 26;14(23):8375. doi: 10.3390/jcm14238375 (PMC12693461; doi:10.3390/jcm14238375)
Supplement: Supplementary file 1 [file jcm-14-08375-s001.zip › jcm-3919222-supplementary.pdf]

Supplementary File S1: COREQ 32-item checklist.

COREQ (Consolidated Criteria for Reporting Qualitative Research) Checklist

Supplementary File S1
Study: *Patient Perspectives after Trapeziectomy versus Carpometacarpal Prosthesis: A Qualitative Thematic Analysis of Ten Bilateral Cases*

Domain / Item	Guide Questions/Description	Reported in Manuscript
Domain 1: Research team and reflexivity		
**1. Interviewer/facilitator**	Which author conducted the interviews?	Methods, 2.3 Data Collection
**2. Credentials**	What were the researcher's credentials?	Methods, 2.3; 2.5 Reflexivity
**3. Occupation**	What was their occupation at the time?	Methods, 2.3
**4. Gender**	Was the researcher male or female?	Methods, 2.3
**5. Experience and training**	What experience/training did the researcher have?	Methods, 2.3; 2.5
**6. Relationship established**	Was a relationship established prior to study commencement?	Methods, 2.3 (no prior treatment relationship)
**7. Participant knowledge of interviewer**	What did participants know about the researcher?	Methods, 2.3 (role explained, not operating surgeon)
**8. Interviewer characteristics**	Any characteristics reported?	Methods, 2.5 Reflexivity
Domain 2: Study design		
**9. Methodological orientation**	What methodological approach was used?	Methods, 2.1 Study Design (thematic analysis Braun & Clarke)
**10. Sampling**	How were participants selected?	Methods, 2.2 Participants
**11. Method of approach**	How were participants contacted?	Methods, 2.2
**12. Sample size**	How many participants?	Methods, 2.2; Results, Table 1
**13. Non-participation**	How many refused/dropped out?	Methods, 2.2 (none reported)
**14. Setting of data collection**	Where was the data collected?	Methods, 2.3
**15. Presence of non-participants**	Was anyone else present besides participants and researcher?	Methods, 2.3
**16. Description of sample**	What are the key sample characteristics?	Methods, 2.2; Results, Table 1
**17. Interview guide**	Was a guide used?	Methods, 2.3; Supplementary File (guide)
**18. Repeat interviews**	Were repeat interviews carried out?	Methods, 2.3 (no)
**19. Audio/visual recording**	Were recordings used?	Methods, 2.3 (audio)
**20. Field notes**	Were field notes made?	Methods, 2.3 (contextual notes)
**21. Duration**	What was the duration of interviews?	Methods, 2.3
**22. Data saturation**	Was data saturation discussed?	Methods, 2.4 (themes stable after 10 cases)
**23. Transcripts returned**	Were transcripts returned for comment/correction?	Methods, 2.4 (member checking, summaries returned)
Domain 3: Analysis and findings		
**24. Number of data coders**	How many coders coded the data?	Methods, 2.4
**25. Description of coding tree**	Did authors provide a description?	Methods, 2.4; Results, Figure/Table 2 (theme overview)
**26. Derivation of themes**	Were themes identified in advance or derived from data?	Methods, 2.4 (inductive)
**27. Software**	What software was used?	Methods, 2.4 (manual coding)

**28. Participant checking**	Did participants provide feedback on findings?	Methods, 2.4 (member checking)
**29. Quotations presented**	Were participant quotations presented?	Results, Sections 3.1–3.4
**30. Data and findings consistent**	Was consistency demonstrated?	Results, 3.1–3.4; Discussion
**31. Clarity of major themes**	Were major themes clearly presented?	Results, Sections 3.1–3.4
**32. Clarity of minor themes**	Were diverse cases or minor themes described?	Results (contrasting views highlighted)

Supplementary File S2: Interview guide (English translation)

Interview Guide – Patient Perspectives after Trapeziectomy vs. Carpometacarpal Prosthesis
(English translation of the original German version used in interviews)

1. Opening / Background
Can you tell me about your experience with thumb arthritis and the operations you underwent?
Which side was treated with trapeziectomy and which with prosthesis?
How long ago were your operations, and in what order did you receive them?
Probes: How did you feel when you were first told you needed surgery? Did you have expectations about the outcomes?
2. Pain and functional recovery
How did you experience pain relief after each surgery?
How long did it take before you could use your hand normally again?
Probes:
Was there a difference in the type or intensity of pain after each procedure?
Did you need pain medication for a longer period on one side compared to the other?
3. Rehabilitation and therapy
How many therapy sessions did you attend after each surgery?
How burdensome did you perceive rehabilitation, exercises, and splinting?
Did therapy interfere with your work, hobbies, or family life?
Probes:
Did you find the instructions from therapists clear and helpful?
Were there differences in motivation or frustration during rehab?
Did you require assistance from family members during recovery?
4. Strength and endurance
How would you compare strength between the two operated thumbs?
Do you notice differences in endurance or fatigue in daily use?
Probes:
Can you carry grocery bags equally with both hands?
Is one hand more reliable for tasks like opening jars, writing, or gardening?
Have you noticed changes in grip strength over time?
5. Daily activities and independence
How well can you perform everyday activities with each hand?
Were there limitations that were more pronounced after one procedure?
Probes:
Impact on housework, cooking, childcare, sports, or professional tasks?
Did either surgery limit your ability to drive, type, or use tools?
How did you adapt when you encountered limitations?
6. Aesthetic perceptions and psychosocial impact
How satisfied are you with the appearance of your thumbs?
Did you notice differences such as shortening, collapse, swelling, or scars?
Probes:
Did appearance influence your confidence in social interactions?
Did you feel the need to hide your hand in public or in photographs?
Did your perception change over time?
7. Long-term experience
How do you feel about each thumb today, years after surgery?
Did either procedure continue to improve or worsen over time?
Probes:
Do you trust the prosthesis to last?
Do you notice increasing weakness or instability on the trapeziectomy side?
Have you ever worried about revision surgery?
8. Overall satisfaction and surgical preferences
What are the main advantages and disadvantages of each procedure from your perspective?
If you had to choose again for a hypothetical “third hand,” which surgery would you prefer and why?
Probes:
Did you feel well-informed before deciding on surgery?

Do you wish your surgeon had told you more about specific outcomes (e.g., aesthetics, strength, recovery time)?
Would you recommend one of the procedures to a friend or family member?

9. Closing / Additional reflections

Is there anything else you would like to share about your experience?
Were there aspects that particularly surprised you or that you had not expected?
Probes:
If you could give advice to surgeons treating future patients, what would it be?
If you could advise another patient considering surgery, what would you tell them?

Supplementary File S3: Coding matrix / theme overview.

Overview of themes, subthemes, and illustrative quotations identified in the analysis

Theme	*Subthemes / Codes*	*Illustrative quotations*
Strength and Function	- Prosthesis perceived as stronger side<br>- Adequacy for daily tasks despite differences<br>- Fatigue more pronounced after trapeziectomy<br>- Long-term reliability vs. gradual decline	"My prosthetic thumb is noticeably stronger." (female, dominant side)<br>"Both sides are sufficient for daily life." (female)<br>"The trapeziectomy side tires faster." (male)<br>"Years later, the prosthesis still feels stable, while the trapeziectomy side keeps losing power." (female)
Rehabilitation and Resilience	- Faster recovery with prosthesis<br>- Reduced therapy burden<br>- Long rehabilitation and coordination training after trapeziectomy<br>- Impact on family and work life<br>- Motivation and frustration during rehab	"After the prosthesis, I needed far less therapy." (male)<br>"Only three therapy sessions, and I was back to normal." (female)<br>"After the trapeziectomy, I had to relearn almost everything." (female)<br>"Therapy after trapeziectomy was exhausting for me and my family." (male)<br>"I was frustrated, because progress was so slow." (female)
Aesthetics and Psychosocial Impact	- Dissatisfaction after trapeziectomy (shortening, collapse, swelling)<br>- Prosthesis perceived as natural appearance<br>- Confidence and willingness to show hands<br>- Impact on social interactions and self-image	"The sunken thumb is aesthetically unappealing." (female)<br>"The prosthesis feels like a normal thumb." (male)<br>"I prefer the prosthetic thumb and hide the other one." (female)<br>"I feel embarrassed showing the trapeziectomy hand when meeting new people." (female)
Surgical Preference and Decision-Making	- Hypothetical choice for prosthesis<br>- Faster recovery and less burden<br>- Reversibility of prosthesis (safety net of trapeziectomy as backup)<br>- Influence of preoperative counseling<br>- Desire for more information about aesthetics and recovery time	"I would choose the prosthesis again without hesitation." (female)<br>"Recovery was significantly shorter with prosthesis." (male)<br>"Why choose a solution with no point of return?" (male)<br>"I wish I had been told more about how my thumb would look after trapeziectomy." (female)
Long-Term Experiences	- Ongoing satisfaction with prosthesis<br>- Perceived decline of trapeziectomy outcomes<br>- Concerns about prosthesis longevity<br>- Awareness of revision options	"I sometimes worry the prosthesis may loosen, but so far it feels strong." (female)<br>"The trapeziectomy side feels weaker every year." (male)<br>"Knowing I could still have a trapeziectomy later makes me less worried." (male)
Quality of Life and Independence	- Faster return to normal activities with prosthesis<br>- Impact on ability to work, care for family, maintain hobbies<br>- Perception of independence and normalcy	"With the prosthesis, I was back to cooking and looking after my grandchildren much sooner." (female)<br>"I can do my gardening again without problems." (male)<br>"The trapeziectomy hand is fine, but I always notice its limits when carrying heavier things." (female)
